# How often parents make decisions with their children is associated with obesity

**DOI:** 10.1186/s12887-018-1283-8

**Published:** 2018-09-25

**Authors:** Adrita Rahman, Kimberly G. Fulda, Susan F. Franks, Shane I. Fernando, Nusrath Habiba, Omair Muzaffar

**Affiliations:** 1 Division of Epidemiology and Community Health, School of Public Health, University of Minnesota, 1300 S 2nd Street, Suite 300, Minneapolis, MN 55454 USA; 2 North Texas Primary Care Practice-Based Research Network (NorTex), University of North Texas Health Science Center at Fort Worth, 3500 Camp Bowie Blvd, Fort Worth, TX 76107 USA; 3 Department of Family Medicine, Texas College of Osteopathic Medicine, University of North Texas Health Science Center at Fort Worth, 3500 Camp Bowie Blvd, Fort Worth, TX 76107 USA; 4 Department of Pediatrics, Texas College of Osteopathic Medicine, University of North Texas Health Science Center at Fort Worth, 3500 Camp Bowie Blvd, Fort Worth, TX 76107 USA

**Keywords:** Obesity, Adolescent – Parent communication, Decision making between parents and adolescents

## Abstract

**Background:**

Evidence supports that better parental involvement and communication are related to reduced obesity in children. Parent-child collaborative decision-making is associated with lower BMI among children; while child-unilateral and parent-unilateral decision-making are associated with overweight children. However, little is known about associations between joint decision-making and obesity among Hispanic youth. The purpose of this analysis was to determine the relationship between parent-child decision making and obesity in a sample of predominantly Hispanic adolescents.

**Methods:**

Data from two studies focused on risk for type II diabetes were analyzed. A total of 298 adolescents 10–14 years of age and their parent/legal guardian were included. Parents completed questionnaires related to psychosocial, family functioning, and environmental factors. Multiple logistic regression was used to determine the association between obesity (≥ 95th percentile for age and gender), the dependent variable, and how often the parent felt they made decisions together with their child (rarely/never, sometimes, usually, always), the primary independent variable. Covariates included gender, age, ethnicity, total family income, and days participated in a physical activity for at least 20 min. ORs and 95% CIs were calculated.

**Results:**

Adolescent participants were predominantly Hispanic *n* = 233 (78.2%), and approximately half *n* = 150 (50.3%) were female. In multivariate analyses, adolescents who rarely/never made decisions together with their family had significantly higher odds (OR = 3.50; 95% CI [1.25–9.83]) of being obese than those who always did. No association was observed between either those who sometimes make decisions together or those who usually did and those that always did.

**Conclusions:**

Parents and children not making decisions together, an essential aspect of parent-child communication, is associated with increased childhood obesity. The results of our study contribute to evidence of parental involvement in decision-making as an important determinant of adolescent health. Further studies should explore temporal relationships between parenting or communication style and obesity.

## Background

Disparities between Hispanic and non-Hispanic populations in the area of childhood and adolescent obesity are critically important to understand, as these may predict related health disparities that can continue throughout life [1–4]. In 2015-2016, 25.8% of Hispanic youth were obese, compared to 22.0% of non-Hispanic black youth and 14.1% of non-Hispanc white youth [5]. Studies have shown that, similar to other ethnic groups [6], the rise in obesity among Hispanic youth is multifactorial involving a combination of genetic factors [7] and environmental factors [1], which include parental influence [8].

Lack of parental involvement and communication have consistently been highly related to obesity in children and adolescents [9–14]. Healthy family functioning, which consists of good communication, problem solving, roles, affective responsiveness, affective involvement, and behavioral control, is associated with more frequent family meals, greater daily vegetable and fruit consumption, more frequent breakfast consumption, fewer hours of sedentary behavior, lower BMI and lower percent overweight in adolescent girls [9]. Greater communication between parents and children also promotes healthier nutritional habits, lower weight and greater physical activity [10–12]. Furthermore, shared parent-child activities have been associated with less overweight and obesity [13]. One study found that children who made more decisions themselves, especially regarding nutrition, were more likely to be obese [14]. Parent-child collaborative decision-making is associated with better health behaviors, including healthy eating behaviors [15].

Unhealthy nutritional habits, physical inactivity, and being overweight or obese are all well-established modifiable risk factors for type II diabetes [16]. Also, having the perception of insufficient parental care and inadequate parental communication has been linked to higher risk for mental and behavioral problems, including unhealthy weight control habits among adolescents [17]. Lower maternal sensitivity is associated with adolescent obesity [18], and poor maternal-child relationships at the ages of 15, 24 and 36 months of age is associated with higher adolescent obesity [19]. Having good communication with parents, therefore, may be a protective factor for obesity and type II diabetes among adolescents.

Children whose parents talk to them about weight loss and restrict their eating practices are more likely to engage in unhealthy and disordered eating habits and gain weight, while those whose parents discuss healthy eating are less likely to eat unhealthy [20–23]. Parents using more lax and coercive disciplinary strategies, fewer health promoting techniques, and possessing less confidence in child lifestyle behavior management are more likely to have obese children [24]. In summation, an authoritative parenting style, where decision-making is collaborative, is associated with lower BMI among children and adolescents [25, 26], while more permissive/indulgent and rejecting/uninvolved parenting styles, where decision-making is child-unilateral, and authoritarian parenting and feeding styles, where decision-making is parent-unilateral, are associated with overweight children [8, 27–30].

Restriction of dietary intake is more common among parents who are racial or ethnic minorities, have low income, and have less than a high school education [31]. However, little is known about associations between parent-child communication and obesity among Hispanic youth. Studies have shown that Mexican-American adolescents have greater respect for parental authority and interdependence and less personal autonomy and independence, indicating less child-unilateral decision making, compared to white American adolescents [32–36]. For instance, Mexican mothers of young teenage daughters expect increases in parent-child mutual decision-making after their daughters turn 15 years old, a delayed age compared to other ethnic groups in the U.S. [36] Furthermore, Mexican-American mothers of very young children are the primary decision-makers when it comes to behaviors related to obesity, including sleep, physical activity and television screen time, although parents and children sometimes or often make decisions together regarding nutrition [37]. There is, however, a lack of a complete understanding of the determinants of the disparities in obesity. For example, participants in focus groups with low-income Hispanic mothers said their children liked fast food, and they placed no restrictions on the food their child wanted and decided to eat [38]. In another study, Hispanic parents said they allowed their child to decide what to eat as alternatives, and pressured them to eat more food [39]. We hypothesized that parent-child cooperative decision making as reported by the parent is associated with childhood obesity in Hispanic and non-Hispanic adolescents.

## Methods

### Study design

The association between parent-child decision making and obesity was explored using data from two cross-sectional studies focused on risk for type II diabetes and adolescence. These studies were titled “Factors Associated with Being at Risk for Type 2 Diabetes among Mexican and Mexican-American Children” (DMMX) and “Psychosocial and Physiological Predictors of Type 2 Diabetes Mellitus among Children Aged 10-14” (PedDM). Data were collected from 298 participants in Tarrant County, Texas between both study protocols. Subjects included adolescents (age 10 to 14 years, male or female, English or Spanish speaking) with a parent or legal guardian. The DMMX study only included Mexican (recruited at a partner institution in Mexico) or Mexican-American (recruited locally in the US) adolescents; whereas, the PedDM study included all race/ethnicities (recruited in the US). Only the Mexican American child participants from the DMMX study were included in the current analysis. The participants recruited in Mexico were not included in this analysis. Identical methods were used for both studies, and participants were recruited from the same geographical area, which allows for combining the data to have a larger sample size. Both studies included nondiabetic child participants. Exclusion criteria from the original studies consisted of having cystic fibrosis, diabetes mellitus, genetic syndromes, hypo- or hyperthyroidism, adrenal disease (Addison’s or Cushing syndrome), taking oral corticosteroids (prednisone, prednisolone, orapred, decadron, dexamethasone) during the past year, or inability to provide consent. Parental consent and child assent were obtained since adolescent subjects were minors. Study procedures included one encounter at the University of North Texas Health Science Center (UNTHSC) that lasted about two hours. Parents completed surveys related to psychosocial, family functioning, and environmental factors. Survey questions were obtained from the National Survey of Children’s Health 2012. Demographic information, such as gender, date of birth, race/ethnicity, socioeconomic status and household size were also obtained. Study materials were available in English and Spanish.

Study methodologies were approved by the Institutional Review Board of UNTHSC at Fort Worth, Texas.

### Dependent variables

The primary dependent variable for this analysis is obesity, a categorical variable. Adolescent participants were classified as obese and non-obese. Body mass index (BMI) was calculated, and participants were categorized into BMI percentiles based on age and gender, according to CDC guidelines [40]. Those who were at the 95th percentile or above were classified as “obese”, and those under the 95th percentile were classified as “non-obese” [41]. BMI was used instead of other measures of obesity since it is routinely collected in a clinic setting.

### Primary independent variables

Parents/legal guardians were asked the question “How often do you feel that your child and you make decisions about his/her life together?” The responses were recorded in a Likert scale as “never,” “rarely”, “sometimes”, “usually” and “always.” The five categories were condensed into four categories; “rarely or never,” “sometimes”, “usually” and “always”. “Rarely” and “never” were combined because there were very few people in the “never” category. This question is used by the Centers of Disease Control and Prevention in the National Survey of Children’s Health, 2007 and the National Survey of Adoptive Parents to assess the subdomain Parent/Child Relationship under Family Functioning [42].

### Covariates

Potential covariates in the current analysis included gender, age, ethnicity (Hispanic, non-Hispanic), total family income per year (less than $10,000, $10,000 to 19,999, $20,000 to $29,999, $30,000 to $39,999, $40,000 or more), and days participated in a physical activity for at least 20 min (less than 7 days, 7 days, I don’t know). The category “I don’t know” was included because the association between the lack of parent’s knowledge regarding their child’s physical activities and the child’s BMI needed to be examined as well as lack of physical activity. It was perceived as representative of the parent’s lack of involvement in the child’s daily activities.

### Statistical analysis

All analyses were conducted using SPSS software version 22 [43]. Descriptive statistics such as means and frequencies are provided for all variables and for levels of the dependent variable BMI (95th percentile or greater and less than the 95th percentile). Independent samples T-tests were used to assess differences between obese and non-obese participants for the continuous variable age, and chi-square tests were used to assess differences in categorical variables between levels of obesity. Simple and multiple logistic regression models were employed to examine associations between obesity and independent variables. Crude and adjusted odds ratios and 95% confidence intervals were estimated. Missing data were excluded from the analysis. Only 2% of cases had missing data. Multi-collinearity between independent variables was tested using Tolerance and Variation Inflation Factor (VIF). Results of the multicollinearity tests showed that collinearity between the variables was very low, with VIF values ranging from 1.005 to 1.023 and Tolerance values between 0.995 and 0.977.

## Results

Table 1 presents the characteristics of the adolescent participants by presence of obesity (BMI equal to or greater than 95th percentile). A total of 298 adolescent participants were included. After missing data were excluded, 292 participants were included in the final multivariate analysis. The adolescent participants were predominantly Hispanic (78.2%) with an average age of 11.9 (SD = 1.4) years. Distribution of gender was essentially equivalent with 50.3% girls. Of participants, 80.5% of parents/guardians reported that they usually or always made decisions with their child. Only 14.9% of adolescents exercised for at least 20 min all seven days of the weeks. One hundred and forty (47.8%) reported a total household yearly income of less than $20,000. Total household income (*p* = 0.04) significantly differed between obese and non-obese adolescents. A majority of youth (52.8%) who live in households with an income of less than $10,000 were obese, compared to a small proportion of obese youth (29.6%) who lived in households with incomes of $40,000 and above.

**Table 1:** Characteristics of the Mexican and Mexican-American Children Study participants by BMI ≥ 95th percentile - Fort Worth, Texas, (*N* = 298)

Variable	Total number (%) of participants for category	BMI ≥ 95th percentile, *n* (%)	BMI <95th percentile, *n* (%)	*p*-value
How often parents make decisions together with child	*n* = 298			0.15
Rarely or never	20 (6.7)	12 (60.0)	8 (40.0)	
Sometimes	38 (12.8)	14 (36.8)	24 (63.2)	
Usually	117 (39.3)	51 (43.6)	66 (56.4)	
Always	123 (41.3)	41 (33.3)	82 (66.7)	
Age, mean (SD)	*n* = 298			0.57
	11.87 (1.405)	11.81 (1.5)	11.90 (1.4)	
Sex	*n* = 298			0.74
Male	148 (49.7)	60 (40.5)	88 (59.5)	
Female	150 (50.3)	58 (38.7)	92 (61.3)	
Ethnicity	*n* = 298			0.17
Hispanic	233 (78.2)	97 (41.6)	136 (58.4)	
Non-Hispanic	65 (21.8)	21 (32.3)	44 (67.7)	
Days of physical activity for at least 20 min	*n* = 296			0.16
7 days	44 (14.9)	12 (27.3)	32 (72.7)	
Less than 7 days	211 (71.3)	90 (42.7)	121 (57.3)	
I don’t know	41 (13.9)	16 (39.0)	25 (61.0)	
Household income	*n* = 293			0.04
Less than $10,000	53 (18.1)	28 (52.8)	25 (47.2)	
$10,000 to $19,999	87 (29.7)	30 (34.5)	57 (65.5)	
$20,000 to $29,999	59 (20.1)	23 (39.0)	36 (61.0)	
$30,000 to $39,999	40 (13.7)	21 (52.5)	19 (47.5)	
$40,000 or more	54 (18.4)	16 (29.6)	38 (70.47)	

*SD* standard deviation

Results of simple logistic regression are shown in Table 2. In bivariate analyses, parent-child decision-making and household income are both significant predictors of obesity. How often youth were reported to make decisions with their parents was significantly associated with obesity. Youth whose parents reported they rarely or never made decisions together were (OR = 3.000; 95% CI [1.137–7.914] more likely to be obese compared to youth whose parents reported they always made decisions together. Additionally, of the covariates, adolescents in households with a total income of less than $10,000 (OR = 2.660; 95% CI [1.201–5.890]) or with a total income of $30,000 to $39,999 (OR = 2.625; 95% CI [1.119–6.155]) were more likely to be obese than those in households with a total income of $40,000 or more.

**Table 2:** Simple logistic regression for BMI ≥ 95th percentile with crude odds ratios

Variable	Crude OR	95% CI
How often parents make decisions together with child		
Always	*…*	*…*
Rarely or never	3.000	(1.137–7.914)
Sometimes	1.167	(0.547–2.490)
Usually	1.545	(0.916–2.609)
Age	0.957	(0.811 - 1.129)
Sex		
Female	*…*	*…*
Male	1.082	(0.680–1.721)
Household Income		
$40,000 or more	…	…
Less than $10,000	2.660	(1.201–5.890)
$10,000 to $19,999	1.250	(0.601–2.600)
$20,000 to $29,999	1.517	(0.693–3.324)
$30,000 to $39,999″	2.625	(1.119–6.155)
Ethnicity		
Non-Hispanic	…	…
Hispanic	1.494	(0.836–2.673)
Days of physical activity for at least 20 min		
7 days	…	…
Less than 7 days vs 7 days	1.983	(0.968–4.064)
I don’t know vs 7 days	1.707	(0.685–4.253)

… = reference group, *OR* odds ratio, *95% CI* 95% confidence interval

Table 3 displays the results of a multiple logistic regression model with obesity as the dependent variable and all other variables as predictors. Adjusting for all other variables, youth whose parents report they rarely or never make decisions together with their parents had significantly higher odds (OR = 3.501; 95% CI [1.247–9.829]) of being obese than those who were reported as always making decisions with their parents. Of the covariates, age, gender, physical activity, and ethnicity had no association with obesity, while household income did. Adolescents living in very low-income households of less than $10,000 (OR = 3.329; 95% CI [1.439–7.703]) and from household incomes between $30,000 and $39,999 (OR = 2.698; 95% CI [1.117–6.515]) had a greater odds of being obese than those who came from families with a household income of $40,000 or greater income even though there were no significant differences between the middle income groups and the highest income group.

**Table 3:** Multiple logistic regression for BMI ≥ 95th percentile with adjusted odds ratios

Variable	Adjusted OR	95% CI
How often parents make decisions together with child		
Always	…	…
Rarely or never	3.501	(1.247–9.829)
Sometimes	1.136	(0.511–2.527)
Usually	1.639	(0.940–2.855)
Age	0.956	(0.800 - 1.144)
Sex		
Female	…	…
Male	1.095	(0.669–1.792)
Household Income		
$40,000 or more	…	…
Less than $10,000	3.329	(1.439–7.703)
$10,000 to $19,999	1.170	(0.551–2.486)
$20,000 to $29,999	1.537	(0.687–3.438)
$30,000 to $39,999	2.698	(1.117–6.515)
Ethnicity		
Non-Hispanic	…	…
Hispanic	1.636	(0.862–3.104)
Days of physical activity for at least 20 min		
7 days	…	…
less than 7 days vs 7 days	2.109	(0.981–4.536)
I don’t know vs 7 days	2.266	(0.852–6.025)

… = reference group; *OR* odds ratio, *95% CI* 95% confidence interval

## Discussion

Parents and children not making decisions together, an essential aspect of parent-child communication, is associated with increased childhood obesity. The results of the present study contribute to evidence of parental involvement in decision-making as an important determinant of adolescent health. In this study, youth whose parents reported they rarely or never made decisions with their parents were more likely to have a BMI in the 95th percentile or above compared to those who always made decisions with their parents. The results complement the findings of studies that support relationships between better parent-child communication and reduced child obesity [8, 28–30, 44].

The significant association found in this study between BMI and how often children are reported as making decisions together with their parents complements the literature. How often adolescents make their life decisions with their parents may be representative of how involved the parents are in their children’s lives, and also how close the parent-child relationship is in terms of communication and trust. Greater parental involvement may lead to children making fewer negative choices, including those regarding their nutritional and lifestyle habits. Better nutritional and lifestyle choices may in turn make them less likely to be obese compared to peers who make unhealthy decisions. Unhealthy nutritional habits include eating disorders, which are associated with perception of low parental caring, poor parent-child communication, and valuing peers’ opinions over parents’ [17]. Therefore, in accordance with previous findings on communication and obesity, adolescents whose parents report rarely make decisions with their families are more likely to be obese.

Interestingly, age does not appear to be a good predictor of obesity in this sample, even though in 2011–2014, there were disparities in obesity prevalence between the age groups of 2 to 5 years, 6 to 11 years and 12 to 19 years [5]. However, the range of our sample is only between the years of 10 and 14 years. Perhaps exploring these associations in a cohort consisting of a wider age range might show different results. Furthermore, the current study did not find gender to be a predictor of high BMI, and there was no statistically significant difference in obesity between Hispanics and non-Hispanics. Being physically active for at least 20 min every day of the week is not associated with decreased obesity in this population, although research shows that physical activity is associated with reduced overweight and obesity among youth [45]. The CDC, however, recommends 60 min of exercise every day for 7 days [46], so perhaps the children in this study were not getting sufficient exercise. A relationship between household annual income of less than $10,000 and presence of obesity is also consistent with the literature, as low socioeconomic status is associated with child obesity. The finding that families earning between $30,000 and $39,999 are more likely to have children with obesity needs further exploration. Results of one study showed that among Mexican-origin families, fathers reported more joint parent-child decision making when they were of high SES, and mothers reported less child-unilateral decision-making when they were of high SES [47]. Despite controlling for the effects of household income, however, a statistically significant association between parent-child decision-making and child obesity remained in our study.

### Strengths

One of the strengths of this study is that weight and height were measured and not self-reported by the subjects. Some studies use self-reported weight and height as opposed to measured weight and height [44, 48]. Although overall self-reported height and weight are positively associated with measured height and weight, females and obese children are statistically more likely to under-report their weight, and children who are shorter than 150 cm are more likely to under-report their height [48]. The BMI percentiles are based on those objective measurements, and the study used the online CDC calculator with age and sex of the child.

### Limitations

A limitation of this study is its cross-sectional nature. This prevents inferring causation between parent-child decision making and child obesity status. Another limitation is that only one component of parent-child decision making is assessed in this study. Additionally, parent-child decision making was measured using a single item. This item has been used by the CDC to measure family functioning in national surveys; however, future research should include a more robust measure. Information about parental obesity, which is positively associated with childhood obesity [49–54], is also not available for this study. The number of children above a BMI percentile of 95 who were reported to have rarely or never made decisions with their parents was also small, leading to wide confidence intervals in our model. Studies should explore this further by recruiting a larger sample of parents who rarely report joint decision-making with their children.

## Conclusions

Future studies should explore temporal or dyadic relationships between parenting or communication style and obesity. Further investigations should explore these associations using causal inference. A longitudinal study would be able to examine these relationships temporally. Those that used self-reported BMI [46] were done in young children, were done in samples not representative of the US youth population [55], or only used maternal relationships [56]. Many cross-sectional studies have been done, but few have been done on how parent-child relationships predict obesity and other cardio-metabolic outcomes later in adulthood. Thus, longitudinal studies should also include cardio-metabolic biological markers in addition to weight and behavioral outcomes.

Additional studies should also include children from different ethnic and cultural backgrounds, as cultural backgrounds could influence relationships between parent-child decision making and obesity in children. For example, a study conducted on Chinese-American youth showed that authoritarian parenting style was associated with lower child obesity, contradictory to studies done on American populations, likely because of greater parental authority and child obedience in Chinese culture compared to American culture [57]. Therefore similar studies should also be conducted with other ethnic populations to see how decision-making is related to weight-related practices and weight status.

Evidence shows that eating behavior can be influenced by sibling behavior [58], and that having an obese sibling increases the likelihood of child obesity [51]. However, most studies investigating parent-child decision making and child weight do not look at sibling relationships, and many that do look at siblings are genetic studies. Therefore, future studies should include relationships between siblings as a potential confounder. One of the limitations was that only one aspect of parent-child communication was explored. Other aspects of communication in relation to obesity status need to be studied. Different developmental ages should be included, as adolescents give more value to their own opinions for making decisions and gradually spend less time with their parents as they grow older [59]. Increasing the age range may help determine when decision-making comes into play and how it affects weight and nutritional health in youth.

## Abbreviations

95% CI: 95% confidence interval
OR: Odds ratio
